# Genomic Correlates of Immune-Cell Infiltrates in Colorectal Carcinoma

**DOI:** 10.1016/j.celrep.2016.03.075

**Published:** 2016-04-14

**Authors:** Marios Giannakis, Xinmeng Jasmine Mu, Sachet A. Shukla, Zhi Rong Qian, Ofir Cohen, Reiko Nishihara, Samira Bahl, Yin Cao, Ali Amin-Mansour, Mai Yamauchi, Yasutaka Sukawa, Chip Stewart, Mara Rosenberg, Kosuke Mima, Kentaro Inamura, Katsuhiko Nosho, Jonathan A. Nowak, Michael S. Lawrence, Edward L. Giovannucci, Andrew T. Chan, Kimmie Ng, Jeffrey A. Meyerhardt, Eliezer M. Van Allen, Gad Getz, Stacey B. Gabriel, Eric S. Lander, Catherine J. Wu, Charles S. Fuchs, Shuji Ogino, Levi A. Garraway

**Affiliations:** 1 Department of Medical Oncology, Dana-Farber Cancer Institute, Harvard Medical School, Boston, MA 02215, USA; 2 Department of Medicine, Brigham and Women’s Hospital, Harvard Medical School, Boston, MA 02115, USA; 3 Broad Institute of MIT and Harvard, Cambridge, MA 02142, USA; 4 Department of Epidemiology, Harvard T.H. Chan School of Public Health, Boston, MA 02115, USA; 5 Department of Nutrition, Harvard T.H. Chan School of Public Health, Boston, MA 02115, USA; 6 Department of Biostatistics, Harvard T.H. Chan School of Public Health, Boston, MA 02115, USA; 7 Division of Gastroenterology, Massachusetts General Hospital, Harvard Medical School, Boston, MA 02114, USA; 8 Clinical and Translational Epidemiology Unit, Massachusetts General Hospital, Harvard Medical School, Boston, MA 02114, USA; 9 Department of Gastroenterology and Hepatology, Division of Internal Medicine, School of Medicine, Keio University, Tokyo 108-8345, Japan; 10 Department of Gastroenterology, Rheumatology and Clinical Immunology, Sapporo Medical University School of Medicine, Sapporo 060-8543, Japan; 11 Division of MPE Molecular Pathological Epidemiology, Department of Pathology, Brigham and Women’s Hospital, Harvard Medical School, Boston, MA 02115, USA; 12 Channing Division of Network Medicine, Department of Medicine, Brigham and Women’s Hospital, Harvard Medical School, Boston, MA 02115, USA; 13 Massachusetts General Hospital Cancer Center, Harvard Medical School, Boston, MA 02114, USA; 14 Department of Biology, Massachusetts Institute of Technology, Cambridge, MA 02139, USA; 15 Department of Systems Biology, Harvard Medical School, Boston, MA 02115, USA

## Abstract

Large-scale genomic characterization of tumors from prospective cohort studies may yield new insights into cancer pathogenesis. We performed whole-exome sequencing of 619 incident colorectal cancers (CRCs) and integrated the results with tumor immunity, pathology, and survival data. We identified recurrently mutated genes in CRC, such as *BCL9L*, *RBM10*, *CTCF*, and *KLF5*, that were not previously appreciated in this disease. Furthermore, we investigated the genomic correlates of immune-cell infiltration and found that higher neoantigen load was positively associated with overall lymphocytic infiltration, tumor-infiltrating lymphocytes (TILs), memory T cells, and CRC-specific survival. The association with TILs was evident even within microsatellite-stable tumors. We also found positive selection of mutations in HLA genes and other components of the antigen-processing machinery in TIL-rich tumors. These results may inform immunotherapeutic approaches in CRC. More generally, this study demonstrates a framework for future integrative molecular epidemiology research in colorectal and other malignancies.

## Highlights

• Whole-exome sequencing of 619 colorectal cancers with clinicopathologic annotations
• Discovery of significantly mutated genes in colorectal cancer
• Neoantigen load correlation with infiltrating lymphocytes and memory T cells
• Positive selection for HLA mutations in immune-cell-infiltrated tumors

## Graphical Abstract

**Figure:**
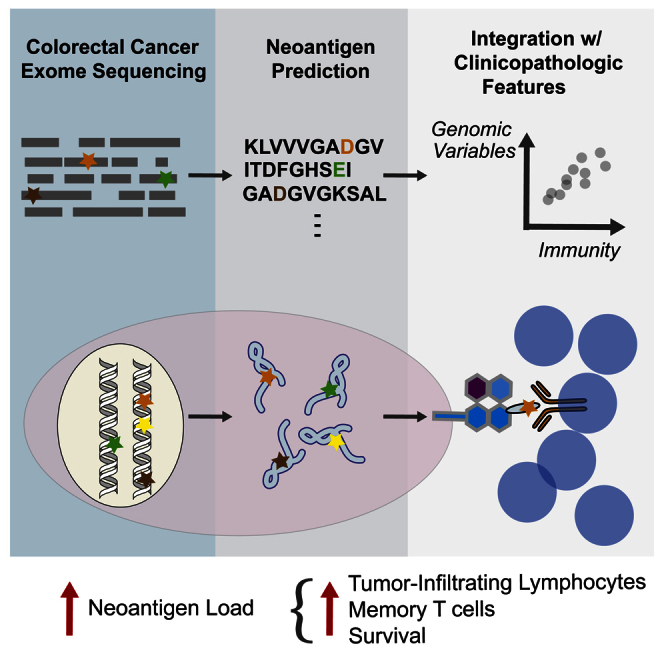

## Teaser

Through whole-exome sequencing of annotated colorectal tumors, Giannakis et al. identify additional colorectal cancer driver genes and correlate high neoantigen load with increased lymphocytic infiltration and improved survival. They also find positive selection for HLA mutations in immune-cell-infiltrated tumors. These results may inform immunotherapeutic approaches in colorectal cancer.

## Introduction

Large-scale cancer sequencing efforts have advanced our understanding of the genomic landscape of many malignancies (Garraway and Lander, 2013). However, a common drawback of comprehensive genomic studies has been the lack of detailed demographic, epidemiologic, and clinical annotations for cancer cases (Cancer Genome Atlas Network, 2012). Together with tumor molecular profiling, such annotations can be used to discover potentially actionable tumor biomarkers, which may change medical practice through lifestyle or pharmacologic intervention (Liao et al., 2012). Another limitation of many tumor sequencing studies has been their limited statistical power to identify significantly mutated genes that have an intermediate or lower frequency of mutation (e.g., <5% frequency). This “long tail” of cancer driver genes can be explored by increasing the number of samples that are sequenced in each tumor type (Lawrence et al., 2014).

Over the past several decades, cancer epidemiologists have invested considerable effort in developing large and exquisitely annotated cohort studies. Two prominent examples include the Nurses’ Health Study (NHS), involving 121,701 women who were followed since 1976, and the Health Professionals Follow-up Study (HPFS), involving 51,529 men followed since 1986. Both studies have accumulated diet, lifestyle, exposure, and survival data on thousands of cancer patients, in conjunction with archived tumor specimens from participants diagnosed with the most common malignancies. These unique annotations have offered insight into various aspects of cancer epidemiology and tumor-environment interactions (Liao et al., 2012, Nishihara et al., 2013).

The integration of genomic data with epidemiologic and pathologic data offers appealing discovery opportunities in colorectal cancer (CRC), a molecularly heterogeneous disease (Cancer Genome Atlas Network, 2012) that arises and progresses in the context of many diverse environmental influences and a complex tumor and immune-cell microenvironment (Ogino et al., 2011). It has been demonstrated that microsatellite instability-high (MSI-high) colorectal carcinomas are associated with high-level immune infiltrates (Jass et al., 1998), that the immune contexture in colorectal carcinomas carries prognostic significance (Galon et al., 2006, Ogino et al., 2009), and that MSI-high tumors respond to immune checkpoint blockade (Le et al., 2015). Across cancers, it has been increasingly recognized that some somatically mutated genes can result in amino acid changes that can generate immunogenic peptides (called neoantigens) and can participate in tumor-specific immune responses (Saeterdal et al., 2001, Schwitalle et al., 2008, Schumacher and Schreiber, 2015). Taken together, these observations suggest a biologically and therapeutically significant interplay between genomics and immune response in CRC. However, few studies have directly examined the totality of neoantigens measured from tumor whole-exome sequencing (WES) in relation to examination of in situ immune-cell infiltration in matched patient samples, and such an analysis has not been performed in microsatellite-stable (MSS) tumors.

To add comprehensive genomic characterization to large-scale epidemiologic cohorts, we performed WES on 619 uniquely annotated archival tumor-normal tissue pairs from the NHS and the HPFS. We identified significantly mutated genes that were previously unappreciated in this disease and linked tumor neoantigen loads and other immune-related genomic aberrations to immune-cell infiltration and prognosis in CRC.

## Results

### Overview of Approach and WES

**Figure 1:**
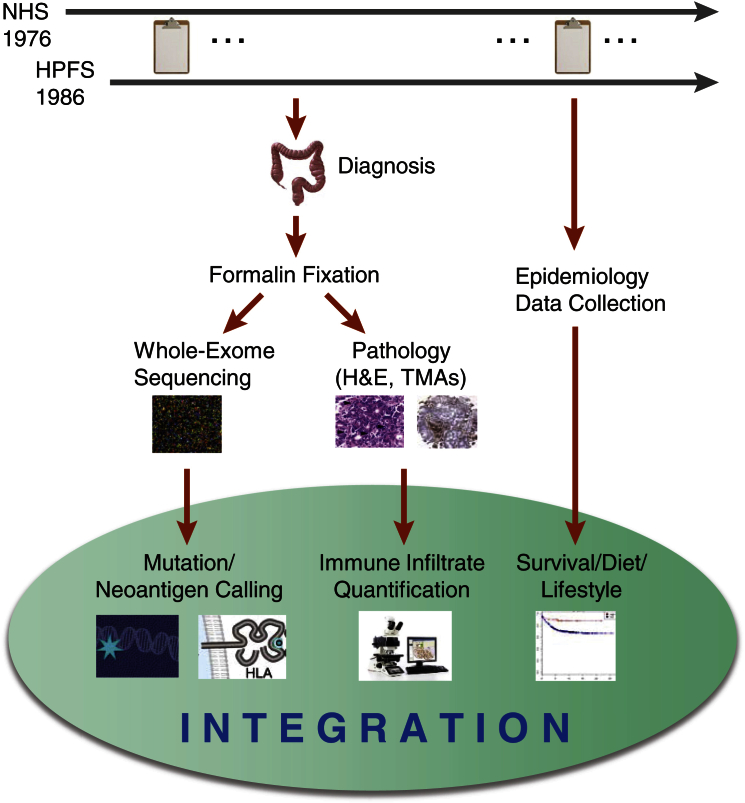
Overview of the Integrative Molecular Epidemiology Approach Employed in the NHS and HPFS Cohorts Participant follow-up used biennial questionnaires. Patients who developed CRC underwent resection of tumor and adjacent normal tissue, followed by formalin fixation, pathologic characterization, and WES.

Our approach for integrating CRC genomic data with pathologic characterization of immune infiltration and clinical outcomes is shown in Figure 1. The NHS and the HPFS represent two U.S.-wide prospective cohorts that have been followed for 40 and 30 years, respectively, by biennial questionnaires (Liao et al., 2012). Throughout the course of follow-up, participants diagnosed with colorectal carcinoma underwent resection of tumor and adjacent normal tissue, which were subsequently fixed in formalin and embedded in paraffin. We constructed tissue microarrays (TMAs) when tissue was sufficient (Chan et al., 2007). These tumor tissues were extensively characterized with pathologic examinations of H&E-stained sections and immunohistochemistry analyses to assess their tumor grade, stage, neoplastic cellularity, percentage of necrosis, and immune infiltrates (Ogino et al., 2009, Nosho et al., 2010).

We performed WES on 619 cases with formalin-fixed paraffin-embedded (FFPE) tumor and matched normal tissue pairs from the NHS and the HPFS (Table S1). The average sequencing coverage across all samples was 90×, with an average of 87% of bases covered at 20× (Figure S1; Table S2). Thus, these results affirm the feasibility of massively parallel sequencing in FFPE tumor samples (Van Allen et al., 2014) and further extend it to decades-old archival specimens. The WES mean target coverage per sample and the library complexity obtained from these archived FFPE tissue blocks inversely correlated with the years since formalin fixation (Spearman’s rank correlation coefficient = 0.33, p value = 5.8 × 10^−30^ for coverage; Spearman’s rank correlation coefficient = 0.48, p value = 6.2 × 10^−69^ for complexity)—but not with the percentage of necrotic tissue within a block, as determined by pathologic examination (Figure S2).

### Significantly Mutated CRC Genes

**Figure 2:**
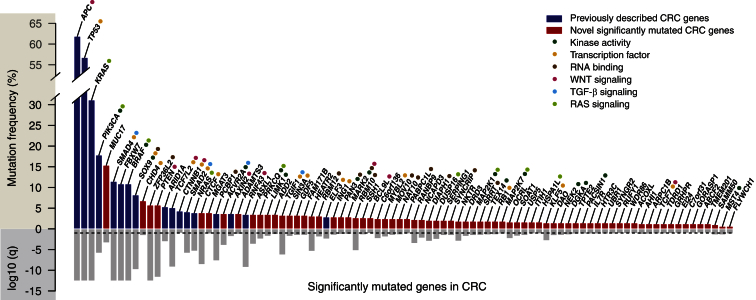
Long Tail of Significantly Mutated Genes in Non-hypermutated CRC Dark blue: genes identified in the NHS and HPFS but also previously reported in other large-scale sequencing studies of CRC (Cancer Genome Atlas Network, 2012, Seshagiri et al., 2012, Lawrence et al., 2014). Red: genes identified in the NHS and HPFS only. Common protein function categories and major CRC signaling pathways are color coded next to significantly mutated genes. Gray bars, lower panel: log10 q value. Dashed line indicates q = 0.1.

Using the somatic mutations, including single-nucleotide variants (SNVs) and small insertions or deletions (indels) detected in the WES data (Table S3), we next identified the recurrently mutated genes that reached statistical significance in this dataset (Figure 2; Tables S4 and S5 for non-hypermutated and hypermutated tumors, respectively). Here, we employed the MutSigCV suite of computational tools (Lawrence et al., 2014), which take into account regional heterogeneity in mutation rates across the genome. In 488 non-hypermutated tumors, we found 90 significantly mutated genes that include most genes observed in the Cancer Genome Atlas Network (2012) analysis of CRC, as well as *RNF43*, which we described to be frequently mutated in this disease (Giannakis et al., 2014). Among these 90 genes, 73 genes were new to CRC in terms of statistical designation as driver genes (Figure 2). This is largely because previous large-scale sequencing studies of CRC either were not powered to detect statistical significance at the requisite mutational frequencies or did not identify the genes (Cancer Genome Atlas Network, 2012, Seshagiri et al., 2012, Lawrence et al., 2014). Putative functions for these genes range from kinase activity to DNA and RNA binding (Figure 2). Pathways critical in CRC pathogenesis (Cancer Genome Atlas Network, 2012), such as WNT, RAS, and transforming growth factor β (TGF-β) signaling, were well represented in the long tail of significantly mutated genes.

The list of cancer genes from this CRC cohort includes known driver genes in other malignancies, such as *PTEN* and *RB1*; WNT signaling genes, such as B cell CLL/lymphoma 9-like (*BCL9L*) and transcription factor 7 (*TCF7*) genes; and RNA processing genes, including *RBM10* and *RBM12*. In addition, we identified recurrent mutations in CCCTC-binding factor (*CTCF*), which co-localizes with cohesins to affect transcription (Katainen et al., 2015); Krueppel-like factor 5 (*KLF5*), a Zn-finger transcription factor that has been implicated in cancer-stem cell regulation (Nakaya et al., 2014); and *TGIF1*, which is a co-repressor of TGF-β signaling. These results demonstrate the potential for gaining insights into CRC pathogenesis by exploring the long tail of significantly mutated genes in CRC.

### Neoantigen Load and Immune-Cell Infiltration

**Figure 3:**
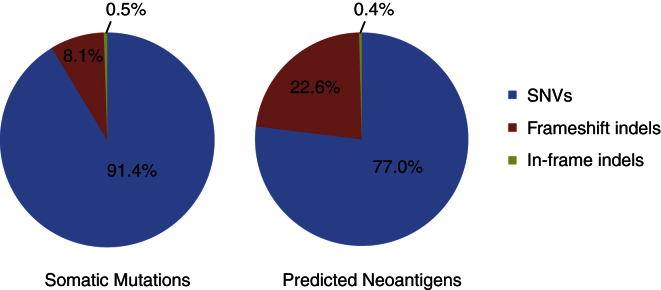
Breakdown of the Total Number of Somatic Mutations and Predicted Neoantigens by Mutation Type

Apart from altering gene function in a way that contributes to carcinogenesis, tumor somatic mutations may affect tumor progression by generating novel peptides that in turn may be recognized by the host immune system as foreign, i.e., neoantigens (Schumacher and Schreiber, 2015), and hence elicit strong anti-tumor immunity. In CRC particularly, immune infiltration has been demonstrated to have prognostic relevance (Galon et al., 2006, Ogino et al., 2009), but the antigenic determinants of this response have not been elucidated. To comprehensively characterize the immunogenic potential of mutations in CRC, we proceeded to predict the number of immunogenic peptides in each tumor sample based on its somatic mutation profiling. We first determined the human leukocyte antigen (HLA) class I type of each sample (Shukla et al., 2015) and then used mutations to predict neopeptides that bind with high affinity (<500 nM) to personal HLA molecules (Rajasagi et al., 2014). Frameshift indels generated a proportionally higher number of neoantigens than did SNVs (Figure 3). We ranked the neopeptides by frequency and found that significantly mutated CRC genes such as *RNF43*, *KRAS*, and *NRAS* contain recurrent neopeptides (Table S6).

We hypothesized that neoantigen load might be associated with the density of immune infiltration in CRC. In 525 tumor-normal pairs that underwent WES, we performed pathologic examination on whole tumor tissue sections and graded each of four components of lymphocytic reactions (on a 0–3 scale) as previously described (Ogino et al., 2009). These include the Crohn’s-like lymphocytic reaction, the peritumoral reaction (discrete lymphoid nodules surrounding the tumor), the intratumoral periglandular reaction (lymphocytes within tumor stroma), and tumor-infiltrating lymphocytes (TILs, defined as lymphocytes on top of cancer cells). The overall lymphocytic score (ranging from 0–12) was composed of the sum of scores for these four components. Overall lymphocytic score, as well as each of the four components, has previously been associated with longer survival of CRC patients in the NHS and HPFS cohorts (Ogino et al., 2009).

**Figure 4:**
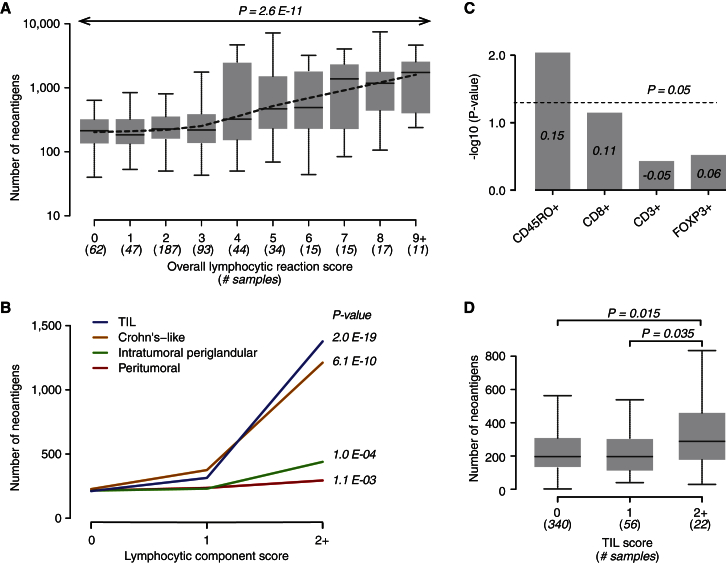
Correlation of Neoantigen Load with Immune-Cell Infiltration in CRC (A) Correlation of neoantigen load with overall lymphocytic reaction score. The dotted line denotes a smoothing curve for all data points. The p value is calculated by Spearman’s rank correlation test. (B) Correlation of neoantigen load with individual components of lymphocytic reaction. The median number of neoantigens for each component score is plotted. The p value is calculated by Spearman’s rank correlation test. (C) Correlation between neoantigen load and density of immunohistochemically defined tumor-infiltrating T cell subsets. Numbers inside bars correspond to the Spearman’s rank correlation coefficient. The p value is calculated by Spearman’s rank correlation test. (D) Comparison of number of neoantigens among tumors with different TIL grades, with analysis restricted to the MSS *POLE*-wild-type subset. The p value is calculated by Wilcoxon rank-sum test.

Integrating these pathologic and neoantigen data revealed that higher neoantigen load was associated with increased overall lymphocytic score in CRC (Spearman’s rank correlation coefficient = 0.29, p value = 2.6 × 10^−11^; Figure 4A). The correlation was also significant at two additional neopeptide affinity cutoffs of 150 and 50 nM (Spearman’s rank correlation coefficient = 0.30, p value = 1.5 × 10^−12^, and Spearman’s rank correlation coefficient = 0.32, p value = 9.3 × 10^−14^, respectively). When we examined the individual lymphocytic reaction components, neoantigen load was most significantly associated with TILs and the Crohn’s-like reaction (Spearman’s rank correlation coefficient = 0.36, p value = 2.0 × 10^−19^, and Spearman’s rank correlation coefficient = 0.27, p value = 6.1 × 10^−10^, respectively; Figure 4B; Table S7). These findings raised the possibility that neopeptides might be recognized by lymphocytes (TILs) that are in immediate contact with tumor cells. Our results may also posit an important role for tertiary lymphoid structures such as the Crohn’s-like reaction in colorectal tumor-directed immune responses.

To identify specific T cell subsets that might correlate with neoantigen load, for 299 samples, we quantified the densities of CD3^+^ (total), CD8^+^ (cytotoxic), CD45RO^+^ (memory), and FOXP3^+^ (regulatory) T cells by leveraging the TMA, immunohistochemistry, and computer-assisted image analysis performed previously on these cohorts (Nosho et al., 2010). We found that the neoantigen load correlated significantly with CD45RO^+^ T cell density but not significantly with CD8^+^, CD3^+^, or FOXP3^+^ T cell density (Figure 4C; Table S7). These results support the idea that prior neoantigen-directed tumor recognition drives the generation of these T cell infiltrates in CRC.

It has been previously shown that MSI-high cancers have increased immune infiltrates, and it has been proposed that MSI-high tumors harbor more neoantigens (Galon et al., 2006, Ogino et al., 2009). We found that MSI-high CRCs and tumors harboring *POLE* exonuclease domain mutations were associated with a significantly higher neoantigen load when compared to MSS cancers (Figure S3). When we restricted our analysis to MSS *POLE*-wild-type cases, we found a significant association between high neoantigen load and high degree of TILs (p value = 0.035 and p value = 0.015 for the comparison of TIL 2+ to TIL 1 and TIL 0 samples, respectively; Wilcoxon rank-sum test; Figure 4D). These findings suggest that even within MSS tumors, neoantigen load is a genomic determinant for immune-cell infiltration in CRC.

### Neoantigen Load and Survival

**Figure 5:**
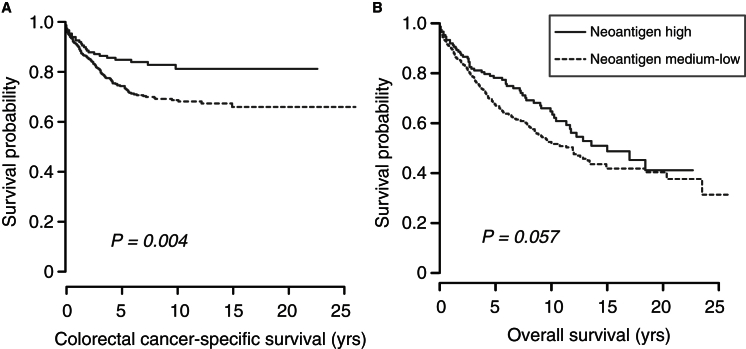
Neoantigen Load and Survival in CRC (A and B) Kaplan-Meier curve for (A) CRC-specific survival and (B) overall survival according to neoantigen load. High (n = 149) and medium-low (n = 448) neoantigen groups were classified as described in Experimental Procedures. The p value is calculated by log rank test.

Previous studies have shown an improved prognosis in CRCs with a high density of memory T cell or cytotoxic T cell infiltrates (Galon et al., 2006, Ogino et al., 2009). Leveraging the extensive clinical data collected on the NHS and HPFS participants, we investigated the relationship between neoantigen load and patient survival. We found that an elevated neoantigen load was associated with improved CRC-specific survival (log rank test, p value = 0.004; multivariate hazard ratio = 0.57 [95% confidence interval, 0.35–0.93], p value = 0.03; Figure 5A; Tables S8 and S9). No significant difference in overall survival was observed (Figure 5B; Tables S8 and S9); this may reflect the large number of non-cancer-related deaths in this population with a median follow-up of 9.4 years (interquartile range, 5.8–13.1 years).

### Positive Selection for HLA and Antigen-Processing Machinery Mutations in Tumors with TILs

Given the relevance of neoantigens in determining immune infiltrates in CRC, we hypothesized that alterations of HLA loci and other antigen-processing machinery (APM) components might undergo positive selection during tumor evolution in the setting of an active antitumor immune response. We identified HLA class I mutations using a computational algorithm that we recently developed (Shukla et al., 2015) and found a total of 96 HLA mutations in 66 of 619 samples (11%). Of these, 27% (18 of 66 samples) had mutations in more than one HLA allele (Figure S4A). All 18 samples with multiple mutations in HLA alleles had the hypermutated phenotype, while 71% (34 of 48 samples) with only a single HLA mutation were hypermutated. The fraction of putative loss-of-function events (nonsense, splice site, and frameshift indels) was comparable between the hypermutated (56%) and the non-hypermutated (57%) groups. The greatest number of mutations was in exon 4, which codes for the T cell receptor (TCR) binding domain (Figure S4B). After normalizing for exon length, exon 4 was still found to be most enriched for mutations, indicating that abrogation of effective T cell interaction is an important mechanism of immune escape in CRC. The selective pressure on the leader peptide domain (encoded by exon 1), peptide binding domains (encoded by exons 2 and 3), and transmembrane domain (encoded by exon 5) was comparable (Figure S4C). No mutations were found in the cytoplasmic tail domain, which is encoded by exons 6, 7, and 8. Of the 39% (37 of 96) mutations in the peptide binding domains, 46% (17 of 37) mutations occurred in positions that come in direct contact with the peptide and would be expected to profoundly affect peptide binding, likely resulting in ineffective presentation of the HLA:peptide complex to the immune system.

**Figure 6:**
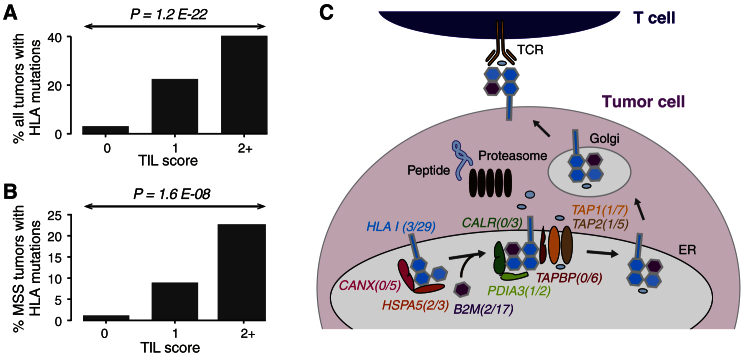
Positive Selection for HLA and APM Mutations in Immune-Cell-Infiltrated Tumors (A) Enrichment of HLA mutations in CRCs with higher TIL scores. The p value is calculated by the chi-square test. (B) Enrichment of HLA mutations in MSS *POLE*-wild-type CRCs with higher TIL scores. The p value is calculated by the chi-square test. (C) Enrichment of APM mutations in immune-cell-infiltrated tumors. Highlighted genes are representative components of the APM pathway, which are mutated in NHS and HPFS tumors. Numbers in parentheses indicate mutation frequencies (percentage) in non-infiltrated versus infiltrated tumors. Neopeptides are processed by the proteasome, transported into the ER, and loaded onto MHCs that, through vesicle transport, traffic to the plasma membrane of tumor cells, where they can be recognized by effector T cells.

**Figure 7:**
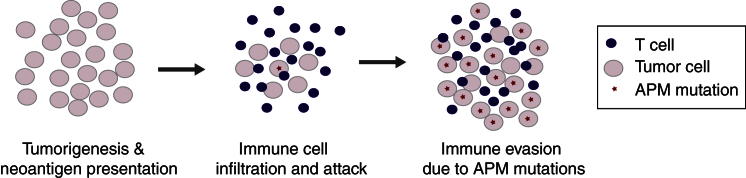
Model of Positive Selection for HLA and APM Mutations in Tumors under Immune Attack

We subsequently correlated HLA mutations with pathologic data of immune infiltration. We observed a significantly higher frequency of HLA mutations in samples with higher TIL grading (Figure 6A; Table S10). This correlation also held when taking into account variations of overall mutation rates among samples and when the analysis was restricted to MSS *POLE*-wild-type tumors (Figure 6B; Table S10). These results suggest that HLA gene mutations may undergo positive selection in tumors with high immune infiltrates. In addition, mutated HLA alleles across all samples were found to have more neoantigens than non-mutated alleles after normalizing for number of coding mutations in the patient (Wilcoxon rank-sum test, p value = 0.006), indicative of a selection process by which the likelihood of an allele being mutated is proportional to its contribution to the tumor’s immunogenicity. This observation supports the hypothesis that acquisition of HLA mutation is a plausible route for escaping immune-based elimination in CRC. We also examined whether, in aggregate, mutations in the APM pathway might be enriched in tumors with high TILs. We found significant enrichment of these mutations in TIL-rich tumors (Table S10). The APM pathway includes proteins involved in major histocompatibility complex class I (MHC class I) folding (CANX and HSPA5), the MHC class I complex (HLA class I and B2M), and the endoplasmic reticulum (ER) peptide-loading complex (TAP, TAPBP, CALR, and PDIA3; Figure 6C). Collectively, these results may indicate positive selection for APM component mutations in tumors that are under immune attack (Figure 7). This may have implications for potential mechanisms of resistance when immunotherapy is administered.

## Discussion

We performed WES on colorectal tumors from two widely studied epidemiologic cohorts and integrated the tumor genomic data with detailed pathologic and clinical information in this setting. Adding to the significant progress in characterizing the molecular landscape of CRC (Cancer Genome Atlas Network, 2012, Seshagiri et al., 2012), new insights were gleaned by exploring the long tail of significantly mutated genes that became evident by sequencing larger numbers of tumors, as predicted by earlier analyses (Lawrence et al., 2014). Newly recognized candidate driver genes include kinases, transcription factors, and RNA binding proteins (Figure 2). Featured in this list were members of the WNT (*BCL9L* and *TCF7*), RAS (*PRKCQ*, *MAP2K1*, and *MAP2K7*), and TGF-β (*TGIF1*) signaling pathways, as well as the RNA processing machinery (*RBM10* and *RBM12*), which were not previously found to be significantly mutated in CRC. BCL9L can act as a co-factor of WNT signaling in tumors and has been shown, when overexpressed, to promote tumor progression in mouse models of WNT-driven colon cancer (Brembeck et al., 2011). The tumor suppressor *PTEN* has been well described as significantly mutated in other malignancies (Lawrence et al., 2014); the increased number of sequenced samples in our study allowed *PTEN* to be identified as such in CRC as well. RBM10 and RBM12 belong to the same family of proteins involved in RNA splicing. Mutations in *RBM10* were described in lung and pancreatic adenocarcinomas and shown to be associated with male sex and an improved prognosis, respectively (Cancer Genome Atlas Research Network, 2014, Witkiewicz et al., 2015). We also identified mutations in reprogramming factor *KLF5*, which regulates the intestinal stem-cell niche and, when deleted, can suppress colorectal oncogenesis (Nakaya et al., 2014). Another transcriptional regulator that was significantly mutated in our analysis was *CTCF*. Our results complement data showing that *CTCF* and cohesin-binding sites are frequently mutated in CRC and positively selected in MSS tumors (Katainen et al., 2015). Our findings demonstrate the potential to gain insight into CRC pathogenesis by exploring the long tail of significantly mutated genes.

In addition to their role in affecting normal cell function, tumor somatic mutations can generate neoantigens, which can be recognized by the host immune system (Schumacher and Schreiber, 2015). A strength of large-scale sequencing analyses is the comprehensive characterization of overall tumor immunogenicity. We identified known CRC genes such as *RNF43*, *KRAS*, and *NRAS* to be the source of recurrent neopeptides, consistent with a prior report (Angelova et al., 2015).

By incorporating the pathologic characterization of the sequenced tumors, we found that neoantigen load was significantly associated with immune-cell infiltrates—including overall lymphocytic score and TILs. Moreover, the concomitant association with CD45RO^+^ T cell subsets argues for the potential importance of memory T cell responses in neoantigen-directed tumor recognition. Supporting this premise, CD45RO^+^ cell density was previously identified as an independent favorable prognostic factor in CRC (Nosho et al., 2010, Pagès et al., 2005). Furthermore, neoantigen load was predictive of CRC-specific survival in our analysis. This result is consistent with literature demonstrating an improved survival in cancers that harbor immunogenic mutations (Brown et al., 2014).

MSI-high CRCs have been shown to contain a higher degree of immune infiltration (Jass et al., 1998); a likely explanation is the presence of many more immunogenic mutations in these tumors compared to their MSS counterparts. Here, analysis of WES data revealed that MSI-high tumors harbored increased numbers of neoantigens; in turn, these were correlated with immune infiltrates. However, even in MSS tumors, neoantigen load was a genomic determinant for immune-cell infiltration in our analyses. In a phase II study, mismatch-repair-deficient tumors were found to respond to checkpoint inhibition, whereas no responses were seen in MSS CRC patients (Le et al., 2015). Our findings suggest that a subset of MSS *POLE*-wild-type tumors has a higher neoantigen load and harbors a higher number of TILs. Such patients may represent an appealing molecular context for future trials of immune checkpoint inhibitors. In addition, although we did not have the statistical power to demonstrate an association with TILs in *POLE*-mutated colorectal tumors, we speculate that they could be responsive to checkpoint inhibition based on their increased neoantigen load.

We also identified and characterized HLA mutations in CRC, demonstrating an enrichment of HLA mutations in the TCR binding domain of the protein. In addition, our results indicate that HLA class I and other APM gene mutations may undergo positive selection in CRCs that exhibit high TIL levels. Thus, APM alterations may provide a mechanism of adaptive resistance to the effects of immune infiltration. Tumor cells that harbor such mutations may exhibit increased evolutionary fitness in the face of immune infiltration, perhaps by impairing their own neoantigen-presentation machinery and thereby shielding themselves from T cells. Whether such tumors might also prove more resistant to immune checkpoint inhibition remains an open question.

In conclusion, we have integrated comprehensive genomic data with clinical, epidemiologic, and pathologic annotations linked to CRC in the NHS and HPFS cohort studies. We found additional putative driver genes and identified neoantigen load as a likely genomic determinant of immune infiltration in CRC. Moreover, HLA and APM pathway mutations show evidence of positive selection in tumors with infiltrating lymphocytes. These results may inform patient selection for future CRC immunotherapy trials. More generally, large-scale genomic characterization of expertly annotated prospective epidemiology cohorts may offer new insights into pathophysiology and therapeutic avenues relevant to multiple cancer types.

## Experimental Procedures

### Specimens and Survival Data

All colorectal carcinoma and adjacent normal colorectal tissue samples were collected according to Partners Human Research Committee institutional review board-approved protocols, and informed consent was obtained from all subjects. Patients were followed until death or January 2012, whichever came first. The National Death Index was used to ascertain deaths of study participants, and survival data were obtained on 597 individuals.

### WES and Identification of Significantly Mutated Genes

Genomic DNA was extracted from tumor areas dissected from tissue sections that were made from the FFPE blocks using the QIAGEN QIAamp DNA FFPE Tissue Kit. We evaluated DNA quality using the Quant-iT Pico Green dsDNAassay Kit (Invitrogen). Sequencing data were generated as detailed previously (Van Allen et al., 2014). In brief, whole-exome capture libraries were constructed from tumor and normal DNA after sample shearing, end repair, phosphorylation, and ligation to barcoded sequencing adaptors. DNA then underwent solution-phase hybrid capture with SureSelect v.2 Exome bait (Agilent Technologies), followed by multiplexing of the samples and sequencing on Illumina HiSeq 2000 instruments. The average coverage was 90× (Table S2). We performed sequence analysis as previously described (Cibulskis et al., 2013, Giannakis et al., 2014). We detected somatic mutations with the MuTect algorithm (Cibulskis et al., 2013) and somatic indels by taking concordant events identified by the Indelocator (http://www.broadinstitute.org/cancer/cga/indelocator) and Strelka (Saunders et al., 2012) algorithms. To further filter spurious SNV calls, we used Burrows-Wheeler Aligner BWA-MEM (http://bio-bwa.sourceforge.net/) to realign sequenced reads associated with the mutations to a set of sequences derived from the human reference assembly. To remove artifacts that result from hydrolytic deamination of cytosine to form uracil specifically in FFPE samples, we filtered out C > T mutations consistent with a 20:1 single-strand bias based on read pair orientation. We used the MutSigCV suite of tools (Lawrence et al., 2014) and manual curation to infer significantly mutated genes. Hypermutated tumors are defined as those with a mutation rate > 12/Mb. All analyses were carried out on human genome build hg19.

### HLA Typing, HLA Mutations, and Neoantigen Load Prediction

The HLA type for each sample was inferred using the POLYSOLVER (POLYmorphic loci reSOLVER) algorithm (Shukla et al., 2015), which employs a Bayesian classifier and selects and aligns putative HLA reads to an imputed library of full-length genomic HLA allele sequences. These alignments are then a basis for the inference step that incorporates the number and base qualities of aligned reads, the empirical library insert size distribution, and the population-based allele frequencies. Mutations in HLA class I genes were detected using the POLYSOLVER-based mutation detection pipeline that takes a tumor-germline exome pair as input and first characterizes the HLA alleles in the individual by applying POLYSOLVER on the germline data. Putative HLA reads from both the tumor and the germline exomes are then aligned to the inferred alleles separately, and likely erroneous alignments are filtered out. Somatic changes are subsequently identified by comparative evaluation of the aligned tumor and germline files using the MuTect (Cibulskis et al., 2013) and Strelka (Saunders et al., 2012) tools.

HLA-binding peptides per sample were identified by a neoantigen prediction pipeline (Rajasagi et al., 2014) that used the somatic mutations detected in an individual, as described earlier. We predicted the binding affinities of all possible 9- and 10-mer mutant peptides to the corresponding POLYSOLVER-inferred HLA alleles using NetMHCpan (v.2.4; Nielsen et al., 2007). To calculate the neoantigen load, we used all predicted binders with affinity < 500 nM, unless otherwise noted.

### Histopathologic Examination and Sample Characterization

H&E slides prepared from FFPE blocks were examined for tumor grade, necrosis, and other tumor characteristics through centralized pathology review (by S.O.). For any given tumor, each of the four components of lymphocytic reaction (Crohn’s-like reaction, peritumoral reaction, intratumoral periglandular reaction, and TILs) was scored as 0 (absent), 1 (mild), 2 (moderate), or 3 (marked), as previously described (Ogino et al., 2009). A subset of CRCs was examined independently by another pathologist (K.I.), and Spearman’s rank correlation coefficient was 0.59 for Crohn’s reaction, 0.67 for peritumoral reaction, 0.56 for intratumoral periglandular reaction, 0.64 for TILs, and 0.68 for the overall lymphocytic score. TMA construction and immunohistochemistry for CD3, CD8, FOXP3, and CD45RO were performed as previously described (Nosho et al., 2010). Immunohistochemically stained TMA slides were scanned by an automated scanning microscope, and Ariol image analysis software was used to quantify the positive nuclei (as the number per square millimeter) in the neoplastic area of each sample. Using DNA from tumor tissue, MSI status was analyzed with the use of 10 microsatellite markers (D2S123, D5S346, D17S250, BAT25, BAT26, BAT40, D18S55, D18S56, D18S67, and D18S487) as previously described (Liao et al., 2012). We used the term MSS for non-MSI-high samples. We used the WES data to identify four MSS samples harboring mutations in the POLE exonuclease domain (p.Pro286Arg, p.Val411Leu, and p.Ser459Phe).

### Statistical Analysis

All p values are two sided. We used the Wilcoxon rank-sum test to compare neoantigen load among *POLE*-mutated tumors, MSI-high tumors, and MSS tumors and used Spearman’s rank correlation and Wilcoxon rank-sum tests to compare neoantigen load and immune infiltrates. Locally weighted scatterplot smoothing was used to calculate the smoothing curve for neoantigen load versus overall lymphocytic reaction score. In survival analyses, we used the Kaplan-Meier method and log rank test, as well as Cox-proportional hazard regression models to calculate the hazard ratio according to neoantigen load, controlling for potential confounders. Specifically, we used a stage-stratified model, adjusting for age, gender, tumor location, and tumor differentiation. We verified that the proportionality assumptions for the Cox model were met. In the analysis of CRC-specific survival, deaths as a result of other causes were censored. Samples were classified as high for neoantigen load if they had more than 450 calculated neoantigens, and remaining samples were classified as medium-low for neoantigen load. This was the optimal cutoff derived from a sensitivity analysis with the division between the high and the medium-low neoantigen groups varying from 200 to 4,000 in neoantigen number with a step size of 50. We performed chi-square test and logistic regression to assess enrichment of HLA and APM mutations in samples with higher TIL scores. We adjusted for mutation rates in logistic regression models for HLA and APM mutations. In addition to HLA, genes participating in the APM pathway were annotated based on the Kyoto Encyclopedia of Genes and Genomes’ (Kanehisa et al., 2014) “Antigen Processing and Presentation Pathway.”

## Author Contributions

M.G., X.J.M., S.A.S., G.G., S.B.G., E.S.L., C.J.W., C.S.F., S.O., and L.A.G. designed research. M.G., X.J.M., S.A.S., Z.R.Q., O.C., R.N., S.B., Y.C., A.A.-M., M.Y., Y.S., C.S., M.R., K.M., K.I., Ka.N., J.A.N., and M.S.L. performed research. E.L.G., A.T.C., Ki.N., J.A.M., E.M.V.A., G.G., S.B.G., and E.S.L. contributed new reagents and analytic tools. M.G., X.J.M., S.A.S., C.J.W., C.S.F., S.O., and L.A.G. analyzed data. M.G., X.J.M., C.J.W., C.S.F., S.O., and L.A.G. wrote the manuscript.

## Accession Numbers

The accession number for all primary sequence files reported in this paper is dbGAP: phs000722.

## Supplemental Information

Document S1. Figures S1–S4

Document S2. Tables S1–S10 Table S1. Clinicopathologic Features of NHS and HPFS Patients and Tumors, Related to Figure 1 Table S2. WES Metrics for NHS and HPFS FFPE Samples, Related to Figure 1 Table S3. Somatic Mutations in the NHS and HPFS Colorectal Cancers, Related to Figure 2 Table S4. Significantly Mutated Genes in Non-hypermutated CRC, Related to Figure 2 Table S5. Significantly Mutated Genes in Hypermutated CRC, Related to Figure 2 Table S6. Recurrent Neopeptides and Associated Genes, Related to Figures 3 and 4 Table S7. Correlation of Neoantigen Load with Components of Lymphocytic Reactions and with Infiltrating T Cell Subsets, Related to Figure 4 Table S8. Baseline Characteristics of NHS and HPFS Patients with CRC, Related to Figure 5 Table S9. Hazard Ratios for Death with Adjustment for Disease Stage and Other Variables, Related to Figure 5 Table S10. Enrichment of HLA and APM Mutations in Samples with Higher TIL Scores, Related to Figure 6

Document S3. Article plus Supplemental Information
