# Supplementary material for: Genomic Correlates of Immune-Cell Infiltrates in Colorectal Carcinoma
Source: Cell Rep. 2016 Apr 14;15(4):857–65. doi: 10.1016/j.celrep.2016.03.075 (PMC4850357; doi:10.1016/j.celrep.2016.03.075)
Supplement: Document S1. Figures S1–S4 [file mmc1.pdf]

Cell Reports, Volume *15*

Supplemental Information

Genomic Correlates of Immune-Cell Infiltrates in Colorectal Carcinoma

Marios Giannakis, Xinmeng Jasmine Mu, Sachet A. Shukla, Zhi Rong Qian, Ofir Cohen, Reiko Nishihara, Samira Bahl, Yin Cao, Ali Amin-Mansour, Mai Yamauchi, Yasutaka Sukawa, Chip Stewart, Mara Rosenberg, Kosuke Mima, Kentaro Inamura, Katsuhiko Nosho, Jonathan A. Nowak, Michael S. Lawrence, Edward L. Giovannucci, Andrew T. Chan, Kimmie Ng, Jeffrey A. Meyerhardt, Eliezer M. Van Allen, Gad Getz, Stacey B. Gabriel, Eric S. Lander, Catherine J. Wu, Charles S. Fuchs, Shuji Ogino, and Levi A. Garraway

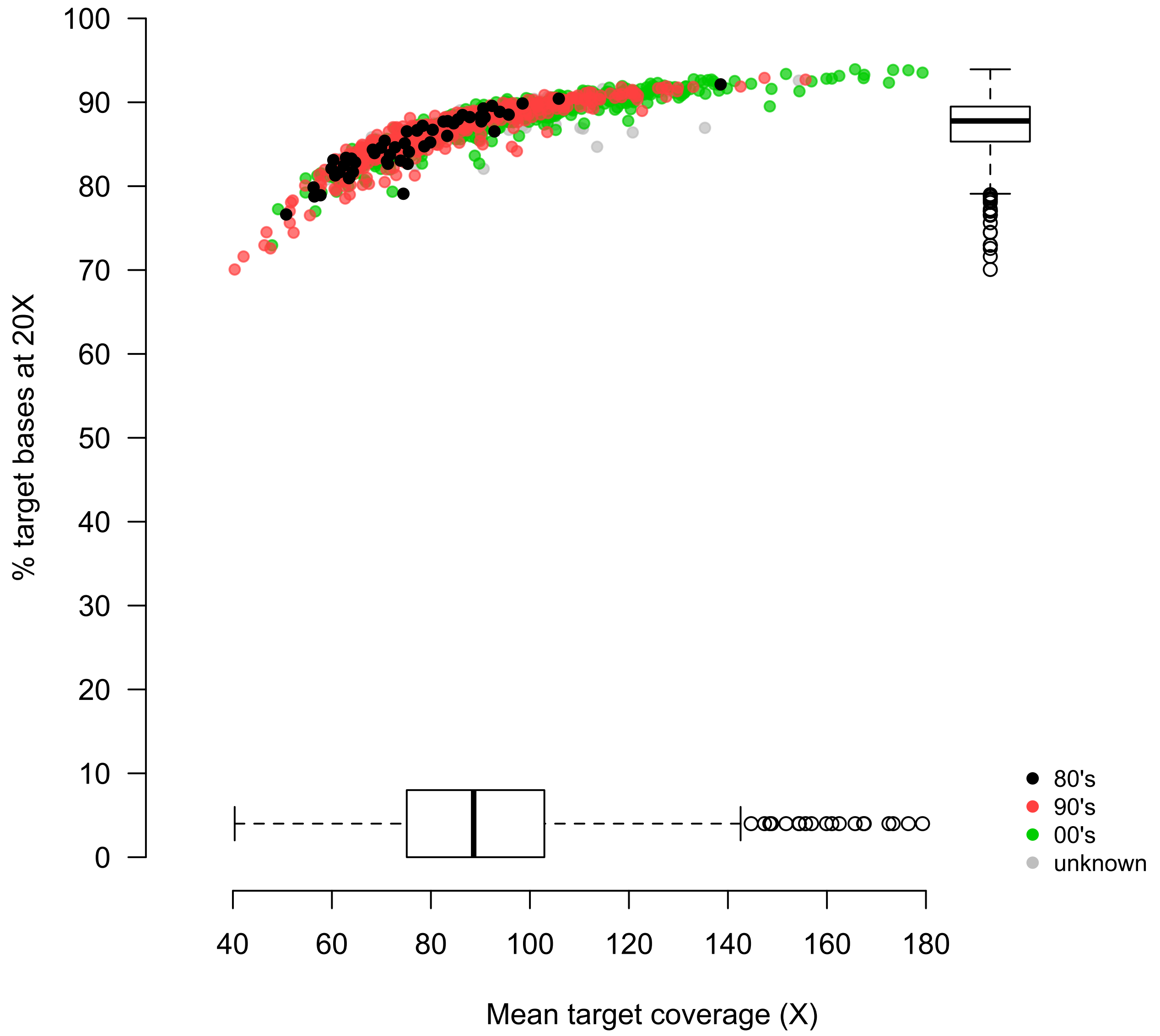

Figure S1, related to Figure 1. Metrics for whole-exome sequencing of 1238 (619 tumor/normal pairs) FFPE samples. Colors indicate the decade in which the sequenced tissues were fixed.

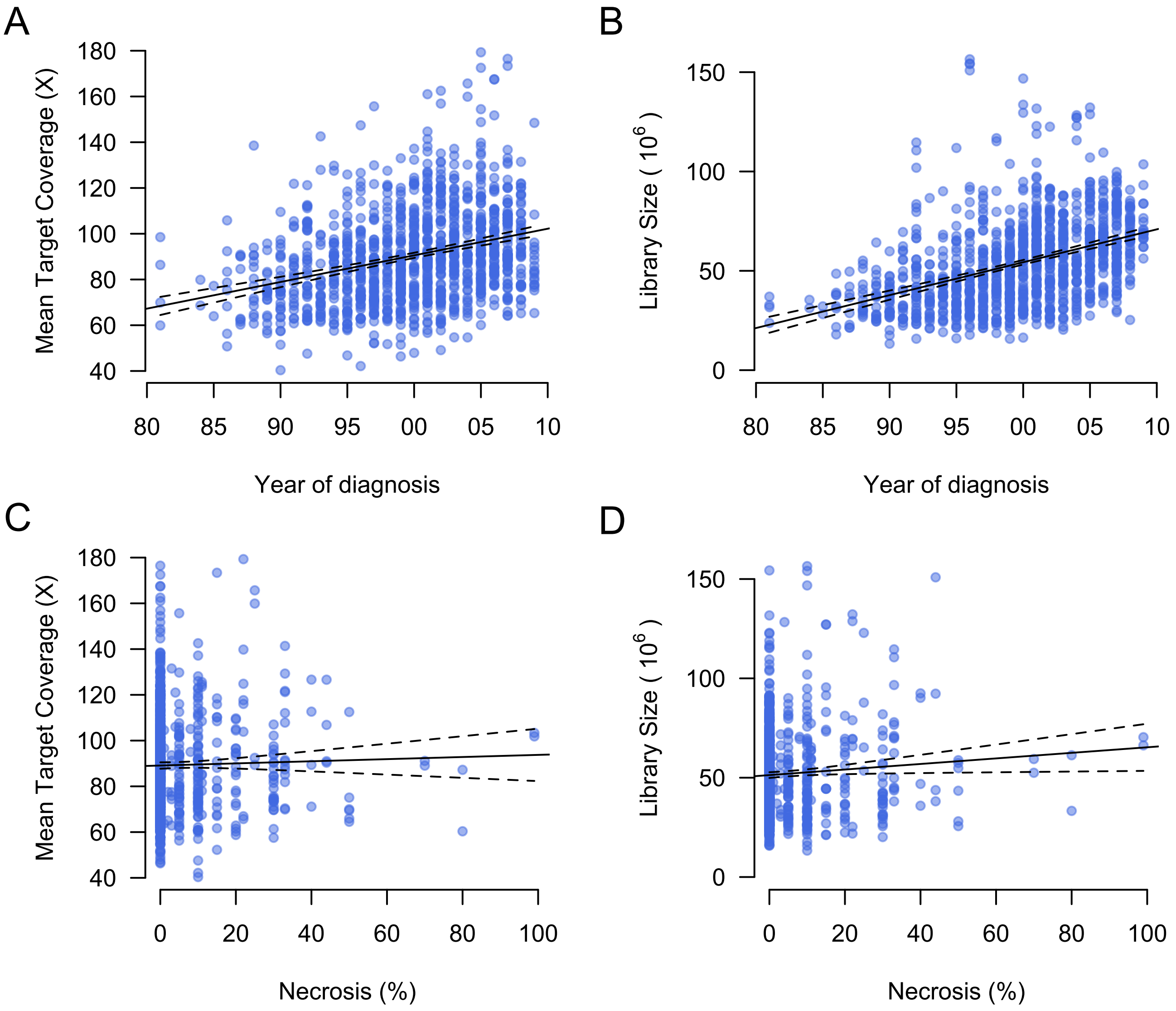

Figure S2, related to Figure 1. Whole-exome sequencing quality inversely correlates with age of tissue block but not with the percentage of tissue necrosis. (A) P-value = 5.8×10^{-30} for mean target coverage versus year of diagnosis, (B) p-value = 6.2×10^{-69} for library complexity (size) versus year of diagnosis, (C) p-value = 0.92 for mean target coverage versus percentage of necrosis and (D) p-value = 0.80 for library complexity versus percentage of necrosis, Spearman's rank correlation.

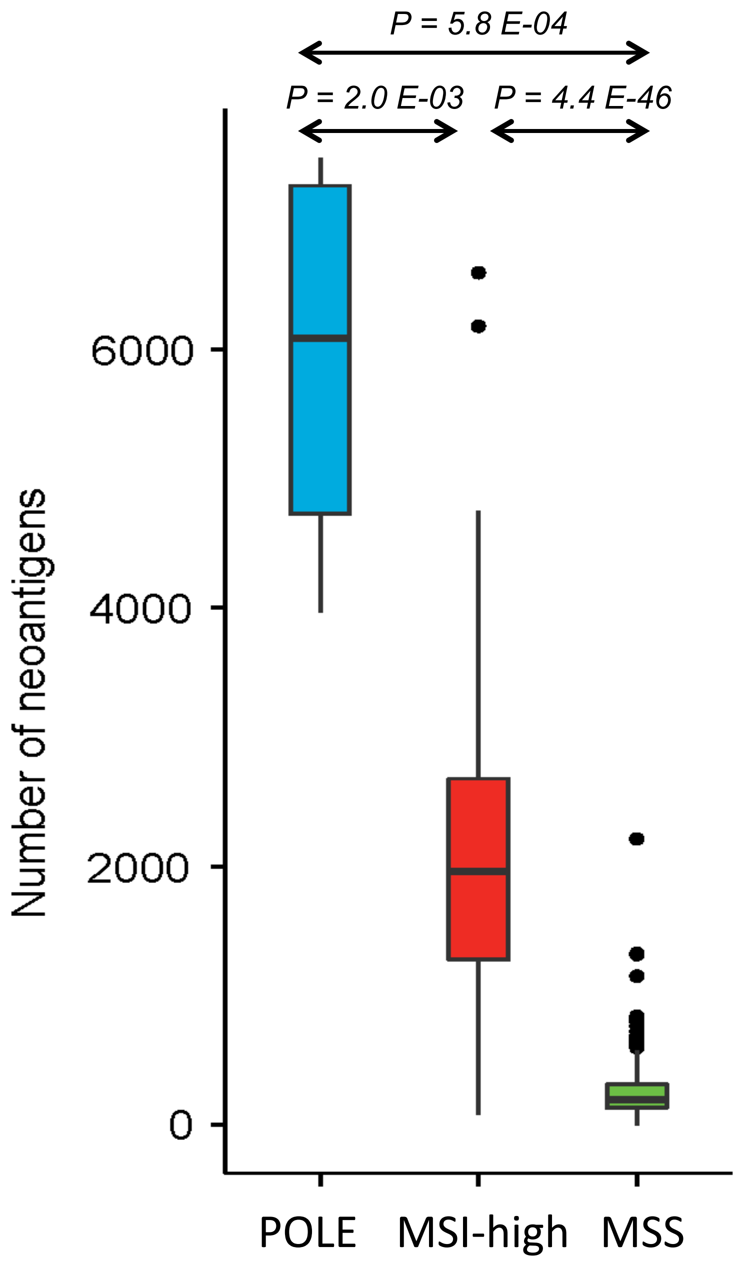

Figure S3, related to Figure 4. Neoantigen load in MSI-high (n=91), MSS (n=434) and *POLE*-mutated (n=4) colorectal cancers. POLE denotes MSS tumors harboring *POLE* exonuclease domain mutations. P-values are calculated by Wilcoxon rank-sum test.

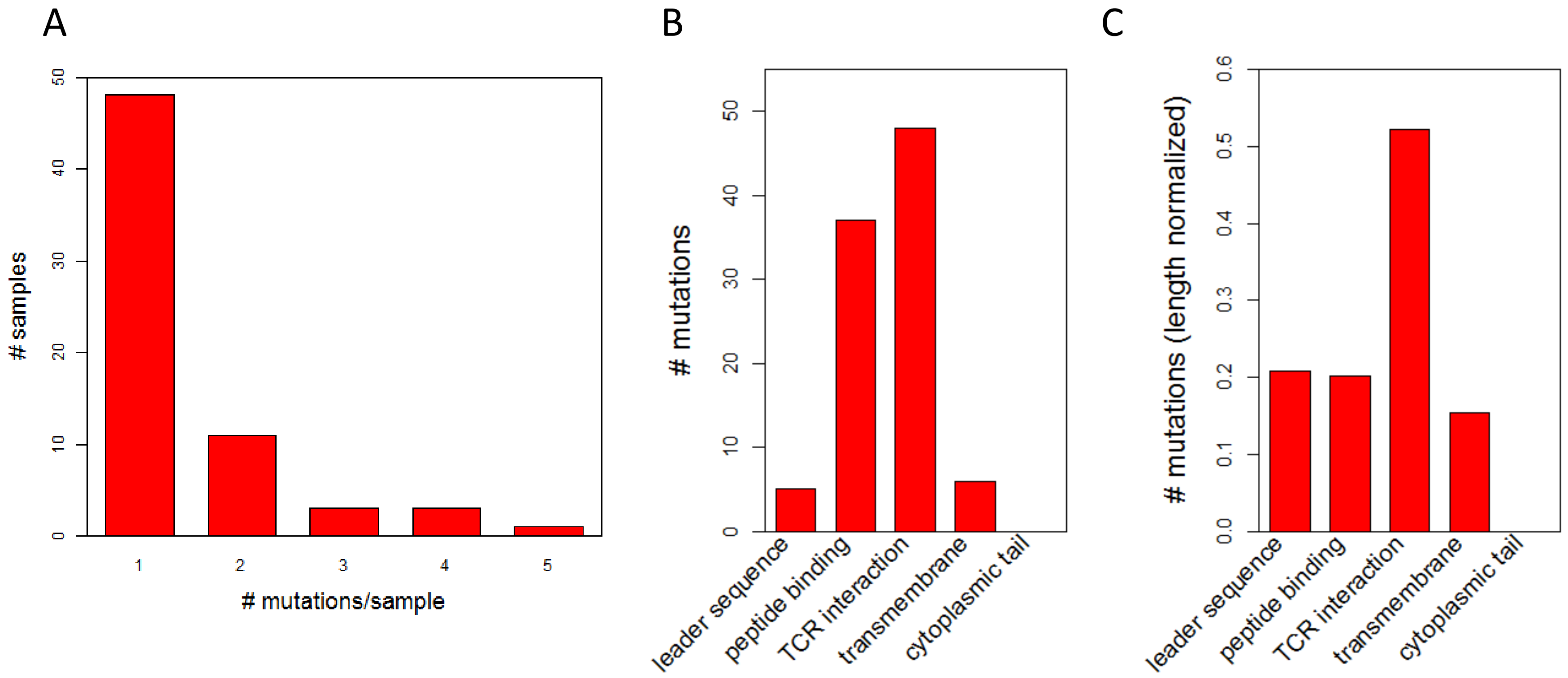

Figure S4, related to Figure 6. Tumor HLA mutations in the NHS and HPFS cohorts. (A) Distribution of HLA mutations among samples. Number of mutations by HLA protein domain, (B) unadjusted and (C) length normalized.
